# Indium-doped ZnO nanowires with infrequent growth orientation, rough surfaces and low-density surface traps

**DOI:** 10.1186/1556-276X-8-493

**Published:** 2013-11-21

**Authors:** Hongfeng Duan, Haiping He, Luwei Sun, Shiyan Song, Zhizhen Ye

**Affiliations:** 1 Department of Materials Science and Engineering, State Key Laboratory of Silicon Materials, Cyrus Tang Center for Sensor Materials and Applications, Zhejiang University, Hangzhou 310027, People's Republic of China

**Keywords:** In-doped ZnO nanowires, Infrequent [02$\bar{2}$3] growth orientation, Large surface-to-volume ratio, Low density of surface traps

## Abstract

Indium-doped ZnO nanowires have been prepared by vapor transport deposition. With increasing In content, the growth orientation of the nanowires switches from [10$\bar{1}$0] to infrequent [02$\bar{2}$3] and the surface becomes rough. No surface-related exciton emission is observed in these nanowires. The results indicate that large surface-to-volume ratio, high free electron concentration, and low density of surface traps can be achieved simultaneously in ZnO nanowires via In doping. These unique properties make In-doped ZnO nanowire a potential material for photocatalysis application, which is demonstrated by the enhanced photocatalytic degradation of Rhodamine B.

## Background

One-dimensional (1D) ZnO nanostructures have attracted extensive research interests in the past decade due to their versatile application potential in nanooptoelectronics [1], electromechanics [2], and catalysis [3]. It has been found that doping impurities, especially group III elements, such as Al [4], Ga [5], In [6], can significantly enhance the electrical conductivity and influence the optical properties. In order to generate desirable electrical, optical, and catalytic properties, 1D ZnO nanostructures have been doped with selected elements. Among these dopants, In is recognized as one of the most efficient elements used to tailor the optoelectronic properties of ZnO [7]. For example, In doping may induce structural defects such as stacking faults [8], twin boundaries [9], and superlattice structures [10], or result in weak localization and electron–electron interactions [11], which can significantly affect the electrical and photoluminescence (PL) properties of ZnO nanostructures. On the other hand, it is quite interesting that In doping can change the morphology of ZnO nanowires (NWs) [12]. There are three typical fast-growth directions ([0001], [10$\bar{1}$0], and [11$\bar{2}$0]) and ± (0001) polar surfaces in wurtzite ZnO [13]. In general, ZnO NWs grow along [0001] direction. When doped with In, however, they may grow along some other directions, such as the non-polar [01$\bar{1}$0] direction [14].

ZnO nanostructures usually have plenty of surface states acting as carrier traps. The existence of such traps is unwanted in catalytic applications, which take advantage of free carriers in the surface region of ZnO nanostructures. In this regard, ZnO nanostructures with large surface-to-volume ratio, high free electron concentration, and low density of surface traps are highly desired.

In this work, we demonstrated that such ZnO nanostructures can be achieved via In doping. The In-doped ZnO NWs were grown by one-step vapor transport deposition. The effect of In doping content on the morphology, structure, and optical properties of the NWs has been investigated. With increasing In doping content, ZnO NWs show switches of the orientation from [10$\bar{1}$0] to an infrequent [02$\bar{2}$3] direction and surface from smooth to ripple-like. Low-temperature PL spectra indicate that indium indeed acts as shallow donor and the density of surface traps is very low. We demonstrated the enhanced photocatalytic performance of In-doped ZnO NWs by degradation of Rhodamine B (RhB) solution.

## Methods

The In-doped ZnO nanowires were synthesized by a vapor transport deposition process in a single-zone high-temperature tube furnace. A mixture of ZnO (99.999%), graphite (99.9%), and In_2_O_3_ (99.99%) powder (weigh ratio 8:2:1) was used as the source material. A layer of 5-nm gold film deposited on the Si (100) substrate before the growth of ZnO NWs was used as catalyst. Then the treated silicon substrate and the source material were placed in a quartz boat and inserted into the tube furnace. Si (100) substrate was placed about 10 cm downstream of the source. Before growth, the quartz tube was evacuated to about 100 mTorr by a rotary pump. Then the tube furnace was heated to 950°C at a rate of 20°C min^−1^, under a Ar flow rate of 100 standard-state cubic centimeter per minute (SCCM). When the temperature reached 950°C, high purity O_2_ was continuously fed into the tube at a flow rate of 2 SCCM, and the pressure was maintained at 4 Torr. After reacting for 30 min at 950°C, the furnace was naturally cooled to room temperature without O_2_ flux, and the white product deposited on the silicon substrate was collected. Undoped ZnO NWs were also grown under the same experimental conditions.

The structure and composition of the samples were analyzed by X-ray diffraction (XRD) through a Rigaku D/max 2550 pc diffractometer (The Woodlands, Texas, USA) and secondary ion mass spectroscopy (SIMS) on a time-of flight mass spectrometer (Ion TOF-SIMS). The morphology and microstructure of the nanowires were characterized by scanning electron microscopy (SEM, Hitachi S-4800, Tokyo, Japan) and transmission electron microscopy (TEM, Philips-FEI Tecnai G2 F30 S-Twin, Hillsboro, OR, USA) combined with selective area electron diffraction (SAED). The In doping content of the individual NW was confirmed by energy dispersive X-ray spectroscopy (EDX) equipped in the TEM instrument. PL spectra were measured on a fluorescence spectrometer (FLS920 Edinburgh Instruments, Livingston, West Lothian, UK), using a He-Cd 325-nm laser as the excitation source.

The photocatalytic activity of the nanowires was evaluated by investigating the photocatalytic degradation of RhB in aqueous solution in a cylindrical quartz photoreactor. Thirty milligrams of each sample was dispersed in 100 ml of deionized water, followed by ultrasonication for 1 h. One milliliter of 1 mM RhB aqueous solution was then added. A Xe lamp was used as the illumination source. Before illumination, the solution was stirred continuously in the dark for 30 min to reach an adsorption-desorption equilibrium of dye molecules on the surface of photocatalysts. The concentration of the remaining dyes was monitored by measuring the absorbance of the solution using a UV–vis spectrophotometer (Shimadzu 3600, Tokyo, Japan).

## Results and discussion

**Figure 1:**
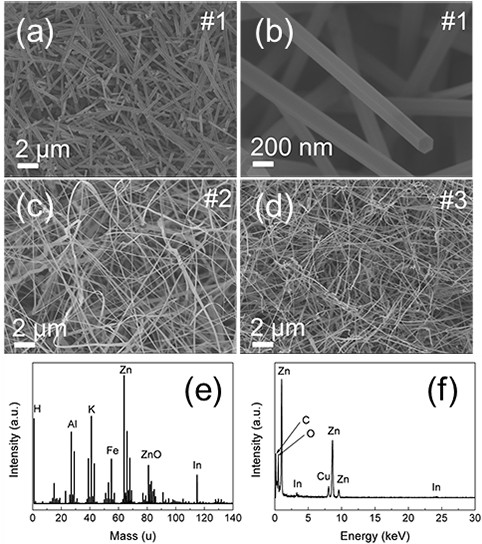
**SEM images and SIMS and EDX spectra. (a)** Low and **(b)** high magnification SEM images of the undoped ZnO nanorods (#1). **(c)** SEM image and **(e)** SIMS spectrum of trace In-doped ZnO NWs (#2). **(d)** SEM image of high content In-doped ZnO NWs (#3). **(f)** EDX spectrum of individual NW in sample #3.

Figure 1a,b shows the low- and high-magnification top-view SEM images of the undoped ZnO nanorods (labeled #1). The sample consists of straight nanorods with uniform diameter of about 200 nm. The uniform hexagonal nanorods are preferentially grown along [0001] direction with smooth surface. Figure 1c,d shows the morphology of the ZnO NWs doped with different In content. It can be seen clearly that the morphology and diameter have changed after In doping. These two samples have similar density and diameter, but the concentration of In dopant are quite different. The In content of the sample showed in Figure 1c (labeled #2) is too low to be detected by EDX, but can be measured by SIMS, as shown in Figure 1e. The ZnO NWs shown in Figure 1d (labeled #3) is heavily doped with In, and the average amount of In in individual NW is about 1.4 at.%, as demonstrated by EDX in Figure 1f.

**Figure 2:**
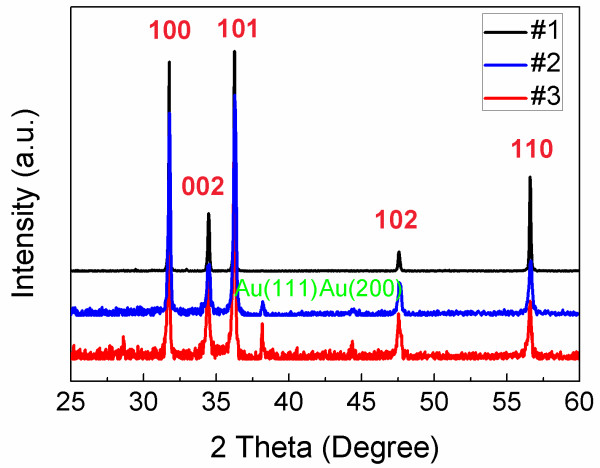
**XRD patterns of ZnO NWs.** Full pattern of undoped (#1) and In-doped (#2, #3) ZnO NWs. No secondary phase is observed in all of the three samples.

X-ray diffraction was carried out to investigate the structure of the three samples. As shown in Figure 2, the patterns reveal that all the samples have hexagonal wurtzite ZnO structure and no extra peak is observed, except the Au (111) and Au (200) peaks, indicating that no secondary phase exists in all of the three samples. The results suggest the successful incorporation of In into ZnO lattice without altering the crystal structure.

**Figure 3:**
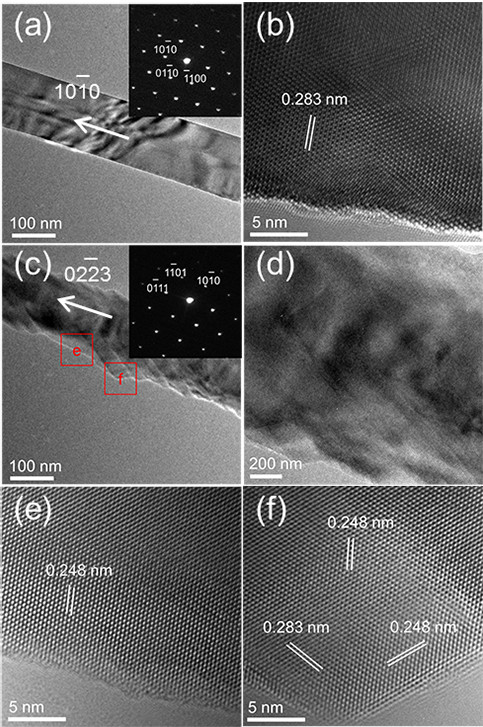
**TEM images and corresponding SAED patterns of In-doped ZnO NWs. (a)** TEM image, **(b)** HRTEM image and its corresponding SAED pattern (inset) of sample #2. **(c,d)** TEM images, **(e,f)** HRTEM images and its corresponding SAED pattern (inset) of sample #3.

In order to further investigate the microstructure of the In-doped samples, TEM and SAED measurements have been carried out over individual In-doped ZnO NW, as shown in Figure 3a,b,c,d,e,f. Significant variation in surface morphology is seen for these two samples. Figure 3a shows the general morphology of the trace In-doped ZnO NWs (#2). It is observed that the NWs in sample #2 have smooth surface with a uniform diameter of about 150 nm. Its HRTEM image (Figure 3b) and corresponding SAED pattern (inset in Figure 3a) reveal a perfect single-crystalline wurtzite ZnO with orientation of [10$\bar{1}$0]. The interplanar distance of fringes is measured to be 0.283 nm, which matches well with the value for (10$\bar{1}$0) planes in wurtzite ZnO. Figure 3c,d shows that the surface of the high-content In-doped ZnO NWs (#3) has ripple-like edges, which is much rougher than that of sample #2, and its diameter is about 150 nm. The HRTEM images of the smooth part (Figure 3e), marked as e in Figure 3c and embossment part (Figure 3f), marked as f in Figure3c, as well as the corresponding SAED pattern (inset in Figure 3c) show a single-crystalline wurtzite ZnO crystal, and reveal that the incorporation of In into ZnO lattice alters the surface morphology of the NWs but not the crystal structure. The interplanar spacing of the planes in the smooth part (shown in Figure 3e) is measured to be 0.248 nm, which corresponds to the spacing of the (0$\bar{1}$11) planes of wurtzite ZnO. But the interplanar spacings of the planes in the embossment part are 0.283 and 0.248 nm which match those of the (10$\bar{1}$0) and (10$\bar{1}$1) planes, respectively. This result indicates that the (0$\bar{1}$11) is the dominant plane, and the NWs mainly grow along an infrequent direction of [02$\bar{2}$3]. As the growth approaches the ripple-like edge, the (10$\bar{1}$0) and (10$\bar{1}$1) facets emerge, and the edge of surface becomes zigzag. Such crystal planes and orientation are not common for ZnO. It is noteworthy that the growth along [0001] direction is suppressed in both of the two In-doped samples. These results definitely indicate that incorporation of In ions into ZnO NWs can promote the tendency of orientation change from the *c*-axis [0001] to an infrequent [02$\bar{2}$3] direction. We believe that the change of preferred orientation is due to the change of surface energy of ZnO planes upon In doping, and the energy difference and relative stability among the (0001), (10$\bar{1}$0), and (0$\bar{1}$11) surfaces vary with increasing doping concentration. Unfortunately, theoretical calculations of the surface energy change are unavailable at this moment. However, it is noteworthy that analogous orientation changes have been observed in Mn-doped ZnO films and testified by the calculation results [15].

**Figure 4:**
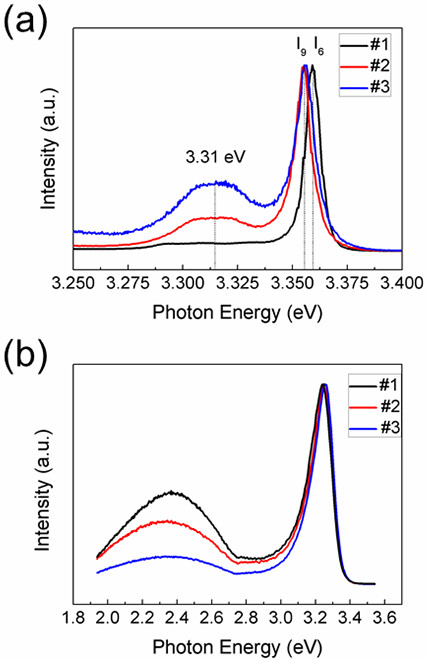
**PL spectra of ZnO NWs. (a)** Low-temperature (14 K) and **(b)** room-temperature PL spectra of undoped (#1) and In-doped (#2, #3) ZnO NWs. The In-doped NWs show donor bound exciton line I_9_ in LT-PL spectra, indicating the formation of In_Zn_ donors.

PL is an excellent method to investigate the impurity and surface states in semiconductors. The optical signature of donor impurities in ZnO has been well established by examining the donor-bound exciton (DBE) emission. On the other hand, due to the large surface-to-volume ratio of ZnO nanostructures, the emission from surface excitons (SX), generally appears around 3.366 eV, has been frequently observed in low temperature PL spectra of many ZnO nanostructures with various morphologies [16-18]. The low-temperature PL (LT-PL) spectra of the three samples at 14 K are plotted in Figure 4a. In the undoped ZnO NWs (#1), the DBE peak locates at 3.360 eV, which corresponds to residual donors, such as Al (I_6_) [19]. In the PL spectra of In-doped ZnO NWs (#2 and #3); however, the DBE peak shifts to 3.357 eV, which is known as I_9_ line and is unambiguously attributed to the exciton bound to In donors [19,20]. This confirms that In is in the substitution site and acts as shallow donor. The emission around 3.31 eV has been a controversial issue for a long time [21-23]. From the low-temperature PL spectra, one can find that the peak around 3.31 eV is observed in both of the two In-doped samples, but not for the undoped one. Furthermore, a direct correlation is found between the intensity of the 3.31 eV emission and the In-doping concentration. Recently, Schirra et al. [21] presented convincing evidences that the 3.31 eV emission in ZnO is related to stacking faults. In our work, the increase of the 3.31 eV emission with In content is consistent with the phenomenon that In doping can easily induce stacking faults in ZnO nanostructures [8]. Therefore, we suggest that the 3.31 eV emission most probably originates from the stacking faults induced by In doping.

From the TEM images (Figure 3c,d), we can observe that the high-content In-doped ZnO NWs have ripple-like surface, which can result in a much larger surface-to-volume ratio and thus facilitate the formation of SXs. Therefore, remarkable surface state-related emission would have been expected in our sample. However, no SX-related emission peak (approximately 3.366 eV) is observed in the low-temperature PL spectrum of sample #3, as shown in Figure 4a. Moreover, the deep level emission, which is found to largely originate from surface defects [24], decreases with increasing In-doping concentration (Figure 4b). These results indicate that the influence of the surface states on the PL properties of sample #3 is almost negligible, which strongly suggests that the density of surface electron traps is at a very low level in our sample.

**Figure 5:**
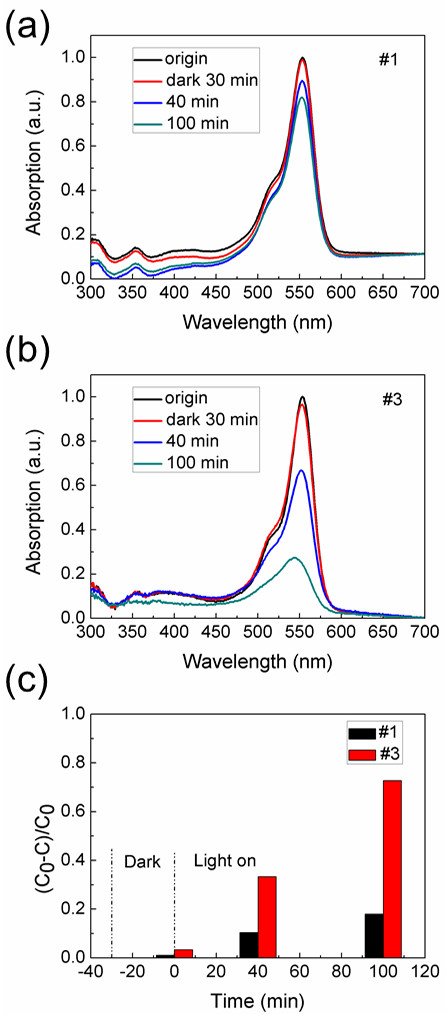
**UV–vis absorption spectra of ZnO NWs.** UV–vis absorption spectral variations of RhB solution over **(a)** undoped and **(b)** In-doped ZnO NWs. **(c)** Degradation rate of RhB solutions over undoped and In-doped ZnO NWs under irradiation.

The realization of ZnO nanostructures with large surface-to-volume ratio and low density of surface traps may enhance the photocatalytic performance. To evaluate the photocatalytic activities of In-doped ZnO NWs, degradation of RhB in aqueous solution was investigated. Figure 5 shows the results of RhB photo-degradation over undoped and In-doped ZnO NWs. It was evident that the ZnO NWs with high In doping content (#3) exhibited much better photocatalytic performance than the undoped one. After illuminating for 100 min, sample #3 was found to degrade nearly 73% of the initial RhB dye, while the degradation over undoped ZnO NWs was less effective, only 20% within the same irradiation time. It is well known that the photocatalytic activities of semiconductor materials are closely related to their morphology, structure and surface properties [25]. Therefore, the much improved photocatalytic performance of In-doped ZnO NWs is probably associated with their large surface-to-volume ratio and low density of surface traps.

**Figure 6:**
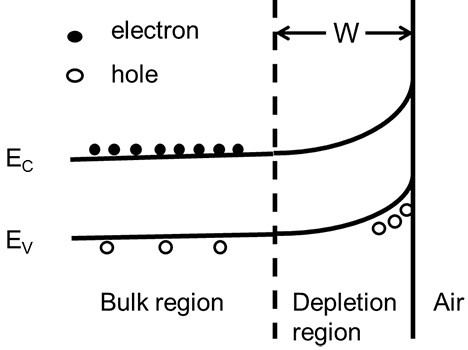
**The schematic of the surface band bending of ZnO NWs.** The energy bands bend upwards as they approach the surface due to the formation of the built-in electric field near the surface, finally results in a surface depletion region and electron–hole separation. Doping of In increases the electron concentration and reduces the width of surface depletion region W, which facilitates the electrons to transport to the surface.

Moreover, high concentration of shallow In donors results in high concentration of free electrons, which will significantly reduce the width of surface depletion region according to the following formula [26]:

(1) $$W=\sqrt{\frac{2\epsilon_{S}V}{eN_{D}}},$$

where *W* is the width of the surface depletion region, *N*_*D*_ is the ionized donor concentration, and *ε*_*s*_ is the static dielectric constant of the semiconductor. The bias *V* depends on the built-in potential *V*_bi_, externally applied voltage *V*_ext_, and *kT*/*e*. As shown in Figure 6, the narrowing of surface depletion region, which would facilitate the electrons to transport to the surface, also contribute to the improvement of the photocatalytic performance.

## Conclusions

In summary, the morphology, microstructure, and PL properties of In-doped ZnO NWs prepared by vapor transport deposition method were investigated. The nanowires exhibit switches of the orientation from [10$\bar{1}$0] to an infrequent [02$\bar{2}$3] direction and the surface from smooth to ripple-like with increasing In doping content. The ZnO NWs with In content of 1.4 at.% have large surface-to-volume ratio with lateral surfaces formed by (10$\bar{1}$0) and (10$\bar{1}$1) facets. Low-temperature PL shows two dominant emissions at 3.357 and 3.31 eV, indicative of the formation of In_Zn_ donors and stacking faults, respectively. The In-doped ZnO NWs do not show surface exciton emission, which indicates a low density of surface electron traps in our samples. We demonstrate that ZnO NWs with large surface-to-volume ratio, high electron concentration, and low-surface trap density can be achieved simply by In doping, which are desirable for efficient photocatalysis.

## Competing interests

The authors declare that they have no competing interests.

## Authors’ contributions

HFD carried out the experiment, measurement, and data analysis and drafted the manuscript. HPH conceived the research, directed the experiment, analyzed the results and revised the manuscript. LWS offered help in experiment and data analysis. SYS performed the PL measurement. ZZY helped in experiments guidance and supervised the project. All authors read and approved the final manuscript.
